# Candida-Induced Emphysematous Gastritis in a Multiple Myeloma Patient: Conservative Management With Favorable Outcome

**DOI:** 10.7759/cureus.18508

**Published:** 2021-10-05

**Authors:** Harshkumar Patel, Faith Buchanan, Christina Chai

**Affiliations:** 1 Internal Medicine, MedStar Washington Hospital Center, Washington, D.C., USA; 2 Internal Medicine/Hospital Medicine, MedStar Washington Hospital Center, Washington, D.C., USA

**Keywords:** portal venous gas, multiple myeloma, intramural gastric air, emphysematous gastritis, candida infections

## Abstract

Emphysematous gastritis is a rare, and often fatal, infection with unclear recommendations on management. We report the first documented case of emphysematous gastritis caused by *Candida* species in a patient on chemotherapy for multiple myeloma. Our patient, who presented with gastrointestinal symptoms was found to have gas in the stomach wall, peri-gastric, and portal veins on CT scan. Nasogastric tube cultures grew *Candida albicans* and *Candida glabrata *and the patient was treated with antibiotics and antifungals. Prompt recognition and conservative management led to a favorable outcome.

## Introduction

Emphysematous gastritis is a rare, potentially lethal infection of the stomach wall caused by gas-forming organisms with mortality approaching up to 60% [1]. While no clear guidelines are available for management, conservative management is the mainstay of therapy, with early initiation of broad-spectrum antibiotics [1-3].

We present a case of an elderly immunosuppressed female on chemotherapy for multiple myeloma who was managed non-surgically for emphysematous gastritis.

## Case presentation

**Figure 1:**
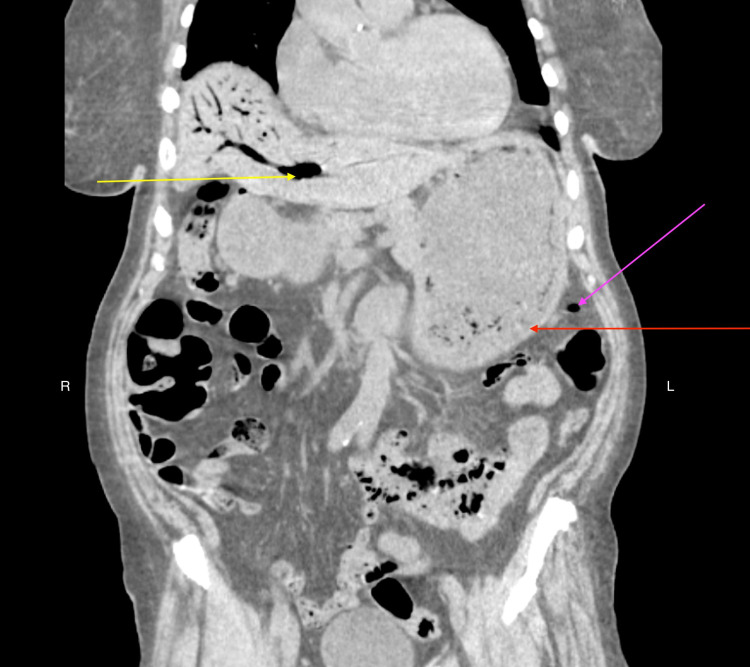
CT scan of abdomen without contrast (coronal view) demonstrating thickened gastric wall with gas bubbles (red arrow), gas in portal venous system - main portal vein, right portal vein, and intra-hepatic veins (yellow arrow), and gas in peri-gastric vein (pink arrow).

**Figure 2:**
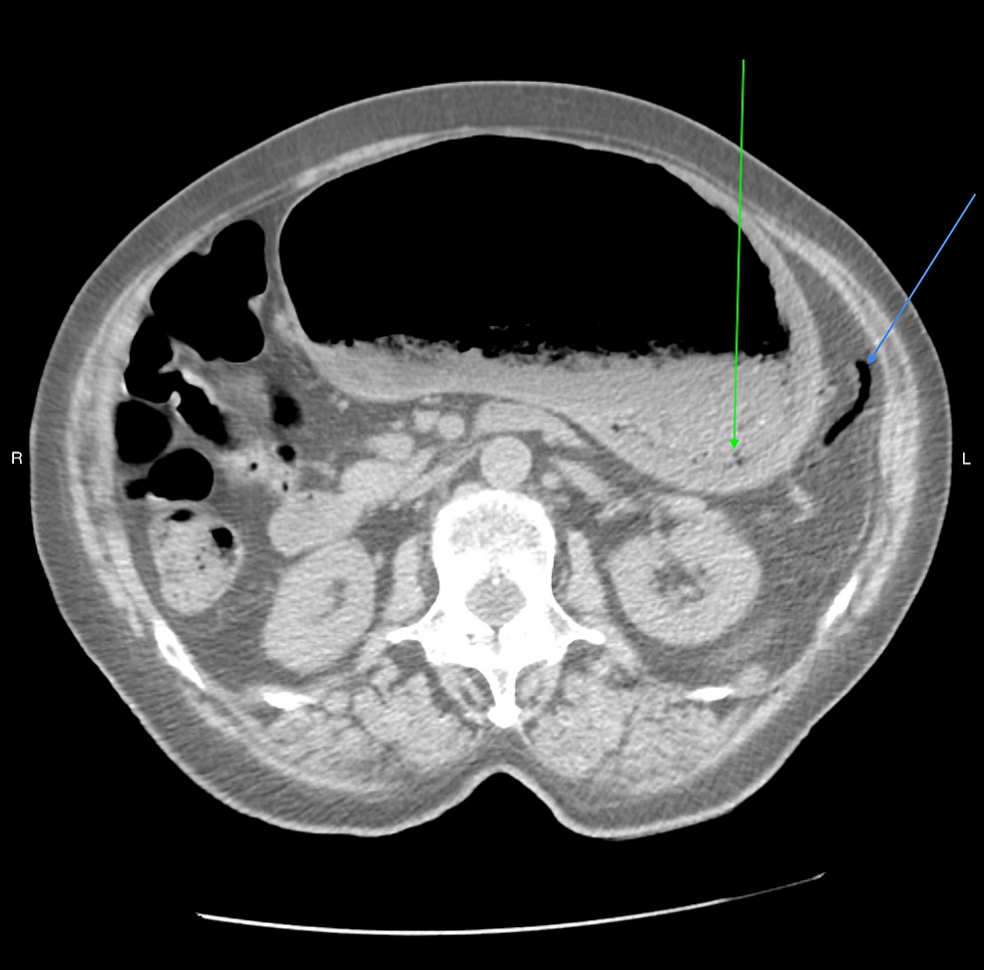
CT scan of abdomen without contrast (axial view) demonstrating distended stomach, thickened gastric wall with gas bubbles (green arrow), and gas in peri-gastric vein along the greater curvature of stomach (blue arrow).

An 81-year-old African American female with a past medical history of relapsed multiple myeloma on elotuzumab, pomalidomide, and dexamethasone (EPD), stage three chronic kidney disease, hypertension, and gastric reflux presented to the emergency room. She was diagnosed with multiple myeloma three years before and her last chemotherapy infusion was 10 days before her presentation to the emergency room. She had no history of gastrointestinal infection, gastrointestinal instrumentation, abdominal surgery, or systemic infection requiring hospitalization after being diagnosed with multiple myeloma. She was on home acyclovir for anti-viral prophylaxis and warfarin for thromboembolism prophylaxis. She complained of progressive nausea, vomiting, abdominal distention, and non-bloody diarrhea of one-day duration. Her vital signs on arrival were temperature of 36.4° C, heart rate of 48 beats/minute, respiratory rate of 17 breaths/min, blood pressure of 111/55 mmHg, and oxygen saturation of 95% on room air. Physical examination was significant for abdominal distention with the presence of bowel sounds and no tenderness to palpation. Initial blood work showed leukocyte count of 8000/μL (4000-10800), hemoglobin of 10.1 gm/dL (11-14.5), platelet count of 198000/μL (145000-400000), BUN of 30 mg/dL (7-23), creatinine of 2.01 mg/dL (0.5-0.8; patient’s baseline around 1.4 mg/dL), sodium of 141 mmol/L (136-145), potassium of 3.8 mmol/L (3.4-4.5), chloride of 105 mmol/L (98-107), calcium of 10.7 mg/dL (8.7-10.4), magnesium of 1.6 mg/dL (1.6-2.6), aspartate aminotransferase of 15 units/L (0-33), alanine aminotransferase of 13 units/L (0-49), and alkaline phosphatase (ALP) of 43 units/L (46-116). The patient underwent a CT scan of the abdomen that showed a markedly distended stomach with gas in the stomach wall, peri-gastric veins, portal venous system - both intra-hepatic and extra-hepatic without pneumoperitoneum, all consistent with emphysematous gastritis (Figure 1 and Figure 2).

**Figure 3:**
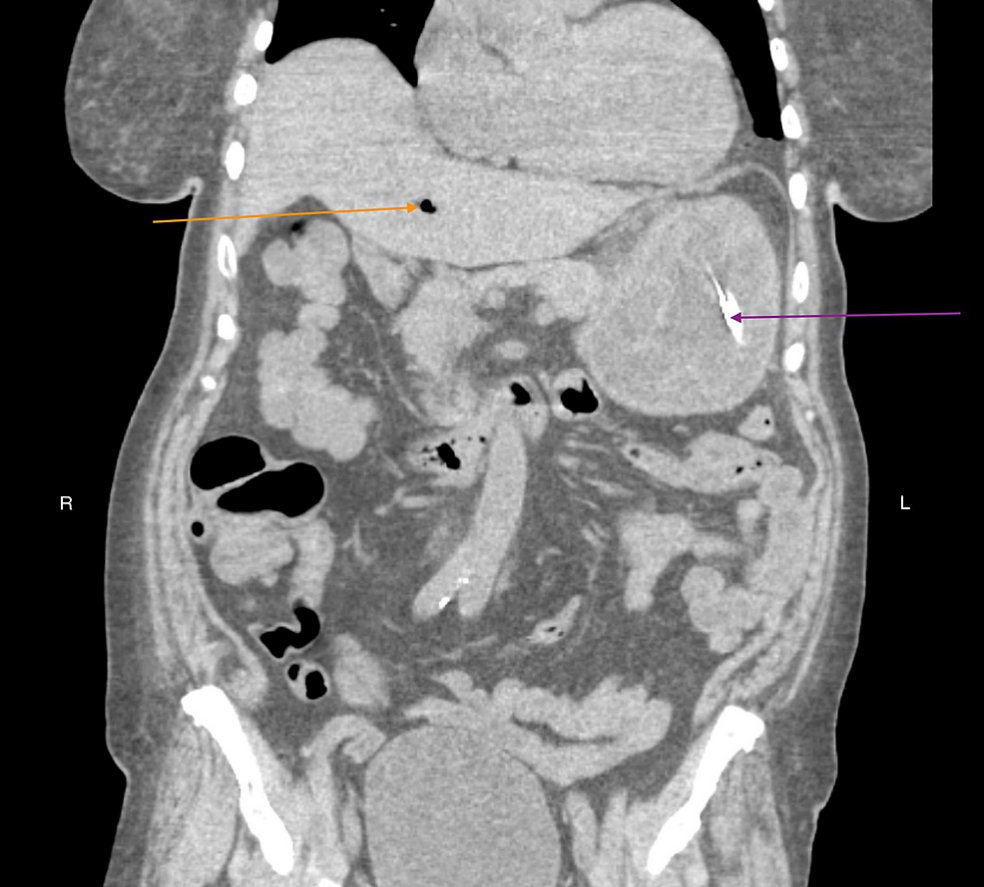
CT scan of abdomen without contrast (coronal view) demonstrating marked improvement in gastric wall edema, nasogastric tube in situ (violet arrow), and minimal gas in main portal vein (orange arrow).

General surgeons recommended conservative management without any surgical intervention. Vancomycin, piperacillin-tazobactam, and micafungin were commenced after blood cultures and nasogastric lavage cultures. She was kept nil-per-os and the nasogastric tube was connected to low continuous suction to relieve abdominal distention per the surgeon’s recommendations. She reported symptomatic improvement with a repeat CT scan two days later showing stomach wall with decreased submucosal edema and only mild pneumobilia (Figure 3).

While blood cultures remained negative, nasogastric lavage cultures grew *Candida albicans* and *Candida glabrata *that were pansensitive. Gastroenterology was consulted but esophagogastroduodenoscopy (EGD) was deferred because of patient preference and symptom improvement. She was commenced on a clear liquid diet on the fifth day of hospitalization and the diet was advanced as tolerated. She was discharged after a 10-day hospital stay with outpatient follow-up with her oncologist. Before discharge, she was switched to oral antimicrobials and completed a 14-day course of anti-bacterial and a 10-day course of anti-fungal treatment based on an infectious disease specialist’s recommendation. She was not recommended to be on anti-fungal prophylaxis due to lack of evidence supporting use in patients with multiple myeloma on EPD. She follows up in the oncology clinic and continues to do well a year after her hospitalization without recurrence of emphysematous gastritis.

## Discussion

Emphysematous gastritis is an uncommon entity that was first described by Fraenkel in 1889; only 59 cases were reported in the English language literature based on a review by Watson et al. in 2017 [4,5]. Emphysematous gastritis is characterized by the presence of air within the gastric wall due to intramural gas-forming organisms and the presence of signs of systemic toxicity that differentiates it from gastric emphysema, which is often a benign finding that carries excellent prognosis [1,6]. Emphysematous gastritis develops in response to either direct or hematogenous infection of the gastric wall [6]. The risk factors include the use of corrosive agents, high alcohol consumption, diabetes, gastric surgery, abdominal surgery, gastric instrumentation, and immunodeficiency [1,7]. The most common pathogens implicated include *Escherichia coli, Enterobacter species, Pseudomonas aeruginosa, Clostridium perfringens,* and *Staphylococcus aureus*, and rarely fungal organisms like *Candida* species [3,8]. Patients with emphysematous gastritis often present with abdominal pain, nausea, vomiting, diarrhea, and signs of hemodynamic instability, with a less dramatic presentation being common in the immunocompromised population [1,5,6,9]. CT scan is the radiologic test of choice and typically shows an irregular, mottled appearance of air within the gastric wall and may show gas in peri-gastric veins, portal veins, or the peritoneum, the latter being a surgical emergency in presence of physical exam findings concerning for peritonitis [3,6,7,9-11]. Culture specimens obtained from gastric mucosa or intra-abdominal fluid collection may help in isolating organisms, and help tailor anti-microbial therapy [1,11]. Our case is the first reported case in the literature of *Candida*-induced emphysematous gastritis in a patient with multiple myeloma. Chemotherapy with EPD is known to increase the risk of infection and we believe it is this that predisposed our patient to develop emphysematous gastritis [12]. EGD may have a role in patients who have clinical deterioration and are being considered for surgical exploration and may help in identifying culprit organisms by providing mucosal samples [13]. EGD findings of inflamed, eroded, or necrotic gastric mucosa are poor predictors for the presence of transmural ischemia and the overall hemodynamic stability of the patient should be considered before proceeding to surgical exploration [13]. While no clear guidelines have been established for the management of emphysematous gastritis, a non-surgical approach with intravenous fluids, early broad-spectrum antibiotics, nil-per-os, and proton pump inhibitors is suggested with surgical interventions reserved for patients with hemodynamic instability, gastric infarction, perforation, or failure to improve with conservative management [1,11,13]. 

## Conclusions

Emphysematous gastritis can have a devastating course and is associated with high morbidity and mortality. Prompt non-surgical management is crucial to prevent dangerous complications like bowel perforation and shock that may require surgical exploration. This case adds to the available data that supports the non-surgical conservative approach. The favorable outcome in our patient was likely due to prompt recognition and management. 
